# Supplementary material for: Validity and reliability of a new whole room indirect calorimeter to assess metabolic response to small calorie loads
Source: PLoS One. 2024 Jun 20;19(6):e0304030. doi: 10.1371/journal.pone.0304030 (PMC11189231; doi:10.1371/journal.pone.0304030)
Supplement: S1 Fig — Observed postprandial change in carbohydrate oxidation (dashed lines) overlaid with Bayesian Hierarchical Model-estimated postprandial change in carbohydrate oxidation (solid lines) for (A) dextrose, (B) fructose, and (C) sucrose beverage conditions. Data are expressed as mean ± standard error of the mean for the observed changes. (DOCX) [file pone.0304030.s001.docx]

**Supplemental Figure 1**

**Observed postprandial change in carbohydrate oxidation (dashed lines) overlaid with Bayesian Hierarchical Model-estimated postprandial change in carbohydrate oxidation (solid lines) for (A) dextrose, (B) fructose, and (C) sucrose beverage conditions. Data are expressed as mean** $\boldsymbol{\pm}$ **standard error of the mean for the observed changes.**

__
