# Supplementary material for: Validity and reliability of a new whole room indirect calorimeter to assess metabolic response to small calorie loads
Source: PLoS One. 2024 Jun 20;19(6):e0304030. doi: 10.1371/journal.pone.0304030 (PMC11189231; doi:10.1371/journal.pone.0304030)
Supplement: S1 Table — (DOCX) [file pone.0304030.s002.docx]

**Supplemental Table 1:** Dietary intake for 24 hours prior to each beverage condition indirect calorimetry session

|  | **Dextrose** | **Fructose** | **Sucrose** | **p-value^a^** |
| --- | --- | --- | --- | --- |
| Total energy intake  (kcal/24 hr) | 2,001 (652) | 2,237 (644) | 2,099 (453) | 0.59 |
| Fat |  |  |  |  |
| Total fat (g/d) | 71.6 (28.1) | 89.2 (28.5) | 83.8 (24.7) | 0.25 |
| Total fat (% kcal) | 32 (4) | 36 (5) | 36 (5) | 0.07 |
| Saturated fat (g/d) | 24.6 (11.0) | 30.8 (12.7) | 26.1 (9.0) | 0.33 |
| Saturated fat (% kcal) | 11 (3) | 12 (3) | 11 (3) | 0.73 |
| Unsaturated fat (g/d) | 40. (17.6) | 51.3 (18.5) | 50.4 (19.0) | 0.28 |
| Unsaturated fat (% kcal) | 18 (5) | 21 (4) | 21 (4) | 0.15 |
| Protein (g/d) | 94.7 (34.0) | 96.0 (31.6) | 104.8 (22.0) | 0.65 |
| Protein (% kcal) | 19 (4) | 17 (3) | 20 (4) | 0.10 |
| Carbohydrate |  |  |  |  |
| Total carbohydrate (g/d) | 245.5 (79.1) | 256.0 (80.7) | 242.0 (61.8) | 0.87 |
| Total carbohydrate (% kcal) | 49 (6) | 46 (5) | 46 (6) | 0.25 |
| Added sugar (g/d) | 47.2 (26.9) | 51.6 (41.8) | 46.9 (21.3) | 0.95 |
| Added sugar (% kcal) | 10 (6) | 9 (6) | 9 (3) | 0.70 |
| Total fiber (g/d) | 22.2 (8.7) | 23.6 (6.3) | 25.8 (8.7) | 0.52 |

^a^ Linear mixed effects model
