# Supplementary material for: Validity and reliability of a new whole room indirect calorimeter to assess metabolic response to small calorie loads
Source: PLoS One. 2024 Jun 20;19(6):e0304030. doi: 10.1371/journal.pone.0304030 (PMC11189231; doi:10.1371/journal.pone.0304030)
Supplement: S2 Table — aMDC, minimal detectable change was calculated as standard error of the mean x 1.96 x √2, where 1.96 is the critical value and √2 is the correction factor for measurement in duplicate. Data are expressed as mean ± standard deviation. (DOCX) [file pone.0304030.s003.docx]

**Supplemental Table 2:** Reproducibility of O_2_ and CO_2_ recovery during infusion validation studies (n=4)

| **Section** | **Error VO_2_ (%)** | **MDC^a^ VO_2_**  **(mL/min)** | **Error VCO_2_ (%)** | **MDC^a^ VCO_2_**  **(mL/min)** |
| --- | --- | --- | --- | --- |
| **1** | 1.33 ± 1.87 | 3.79 ± 1.86 | 2.68 ± 1.38 | 3.29 ± 1.63 |
| **2** | -0.67 ± 1.66 | 1.73 ± 0.29 | 1.11 ± 1.13 | 1.35 ± 0.08 |
| **3** | -1.43 ± 1.06 | 4.34 ± 0.22 | -0.13 ± 1.52 | 3.47 ± 0.54 |
| **4** | -1.35 ± 1.02 | 1.71 ± 0.14 | 0.39 ± 1.46 | 1.27 ± 0.12 |
| **5** | -1.71 ± 1.01 | 2.68 ± 0.5 | -0.33 ± 1.84 | 2.09 ± 0.38 |
| **6** | -2.1 ± 1.19 | 2.52 ± 0.16 | -0.57 ± 1.95 | 2.47 ± 0.42 |
| **7** | -1.65 ± 0.93 | 1.95 ± 0.31 | 0.2 ± 1.95 | 1.3 ± 0.7 |
| **8** | -1.84 ± 1.15 | 1.1 ± 0.38 | -0.14 ± 2.17 | 0.81 ± 0.2 |
| **Total** | -1.29 ± 1.19 | 0.83 ± 0.04 | 0.36 ± 1.57 | 0.69 ± 0.07 |

^a^MDC, minimal detectable change was calculated as standard error of the mean x 1.96 x $\surd2$, where 1.96 is the critical value and $\surd2$ is the correction factor for measurement in duplicate.

Data are expressed as mean ± standard deviation.
