# Supplementary material for: Validity and reliability of a new whole room indirect calorimeter to assess metabolic response to small calorie loads
Source: PLoS One. 2024 Jun 20;19(6):e0304030. doi: 10.1371/journal.pone.0304030 (PMC11189231; doi:10.1371/journal.pone.0304030)
Supplement: S1 File — (DOCX) [file pone.0304030.s004.docx]

Supplemental Materials and Methods

Whole Room Indirect Calorimeter

*Metabolic Chamber Description (additional)*

The chamber has the capability of being operated in either “push” or “push-pull” modes. When operating in “push-pull” mode, an air blower provides a vacuum on the outflow MFC, which regulates pressure inside the chamber. This allows for control of both inflow and outflow rates so that the room can be operated with a lower ventilation rate and at a minimal pressure difference.

The O_2_ analyzer has a constantly flowing reference from a gas tank (~21% O_2_, balance N_2_). The CO_2_ analyzer has a sealed reference cell filled with N_2_. Perma Pure dryers (Perma Pure, LLC, PD-50T-48MSS) remove water vapor from sample gases prior to entering outflow analyzers, and humidity sensors (Viasala, HMP60C12A0A3B0) verify adequately dried samples. In addition, temperature (Vaisala, HMP60C12A0A3B0), humidity (Vaisala, HMP60C12A0A3B0), and pressure (Omega, PX653-2.5BD5V) inside the WRIC are monitored by specific sensors.

*Routine Calibrations*

After each O_2_ analyzer reference gas tank change, a hardware calibration is performed on the O_2_ analyzer, in which the absolute measurement range of the analyzer is established.

Following the hardware analyzer calibration, a blender calibration is performed to ensure linearity of the O_2_ analyzer. During the blender calibration process, a gas blender is used to gflow O_2_, CO_2_, and N_2_ through the full range of the analyzers. Linearity of the O_2_ analyzer readings is then verified (r^2^>0.9999), and differences between known and measured gas concentrations are then calculated to be applied as corrections to VO_2_ and VCO_2_ data collected during experiments. While the CO_2_ analyzer is not inherently linear, linearity of this analyzer is also verified (r^2^>0.999).

*Bi-Annual Maintenance*

All high flow and blender mass flow controllers (MFCs; Alicat Scientific) are calibrated bi-annually by MEI, Ltd. using positive displacement primary piston provers specific to each MFC (ML-800 and ML-1020, Mesa Labs). In the calibration process, each MFC is first tested at 10 points across the full flow range, beginning at 10%; a second test across the full range is then run at 10% intervals beginning at 5%. Each MFC must measure within 0.5% of the prover to pass calibration. All blender MFCs are calibrated using their respective gasses (i.e., N_2_, O_2_, and CO_2_).

Human Study Design

*Control of Dietary Intake*

During a consent visit, estimated energy needs were calculated for each participant using the Mifflin-St. Jeor equation multiplied by an activity factor based on self-reported exercise and physical activity patterns (Mifflin et al., 1990). Participants were then instructed to consume a specified meal (50% of energy from carbohydrate, 30% from fat, and 20% from protein) meeting 35% of their estimated energy requirements between 6-10pm the evening before each session. Foods included in the meal were individualized to participant preferences but contained the same macronutrient composition.

When participants arrived at the laboratory for each experimental session, a 24-hour dietary recall for the previous day was collected using a multiple pass method (Conway, et al., 2003; Conway, et al., 2004); this recall included the specified meal planned during the consent visit. Dietary recalls were analyzed using Nutrition Data Systems for Research (version 2020).

References

Mifflin MD, St Jeor ST, Hill LA, Scott BJ, Daugherty SA, Koh YO. A new predictive equation for resting energy expenditure in healthy individuals. Am J Clin Nutr. 1990;51: 241–247.

Conway JM, Ingwersen LA, Vinyard BT, Moshfegh AJ. Effectiveness of the US Department of Agriculture 5-step multiple-pass method in assessing food intake in obese and nonobese women. Am J Clin Nutr. 2003;77: 1171–1178.

Conway JM, Ingwersen LA, Moshfegh AJ. Accuracy of dietary recall using the USDA five-step multiple-pass method in men: An observational validation study. Journal of the American Dietetic Association. 2004;104: 595–603. doi:10.1016/j.jada.2004.01.007.
